# Supplementary material for: Photocatalytic and Oxidative Synthetic Pathways for Highly Efficient PANI-TiO2 Nanocomposites as Organic and Inorganic Pollutant Sorbents
Source: Nanomaterials (Basel). 2020 Feb 29;10(3):441. doi: 10.3390/nano10030441 (PMC7153600; doi:10.3390/nano10030441)
Supplement: Supplementary file 1 [file nanomaterials-10-00441-s001.pdf]

Supplementary Materials

Photocatalytic and Oxidative Synthetic Pathways for Highly Efficient PANI-TiO_2 Nanocomposites as Organic and Inorganic Pollutant Sorbents

Carolina Cionti [1,2], Cristina Della Pina [1,3], Daniela Meroni [1,2,*], Ermelinda Falletta [1,3,*] and Silvia Ardizzone [1,2]

[1] Dpt. of Chemistry, Università degli Studi di Milano, via Golgi 19, 20133 Milano, Italy.
[2] Consorzio INSTM, via Giusti 9, 50121 Florence, Italy.
[3] ISTM-CNR, via Golgi 19, 20133 Milano, Italy.
* Corresponding authors: daniela.meroni@unimi.it, ermelinda.falletta@unimi.it

Table S1. Composition of the adopted simulated drinking water.

Simulated Drinking Water	
**Ca^{2+} (mg L^{-1})**	45
**Na^{+} (mg L^{-1})**	46
**Mg^{2+} (mg L^{-1})**	9
**Cl^{-} (mg L^{-1})**	79
**SO_4^{2-} (mg L^{-1})**	37
**HCO_3^{-} (mg L^{-1})**	122
**conductivity (µS cm^{-1})**	478
**pH**	7.0

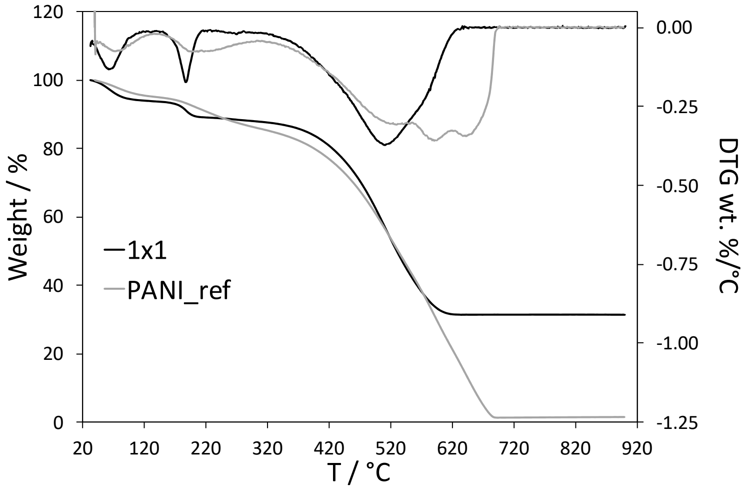

Figure S1. TGA and DTG analyses of 1 × 1 composite compared to reference PANI_ref.

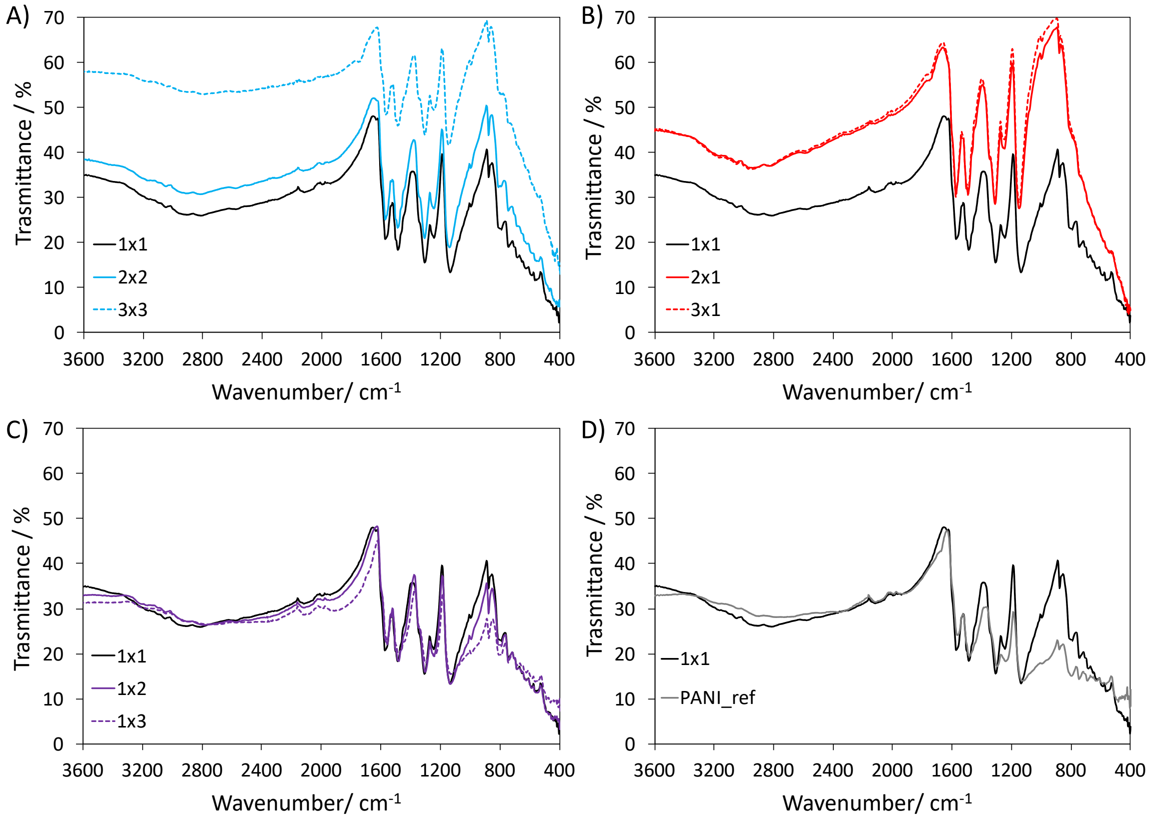

Figure S2. FTIR spectra of 1x1 composite compared to **A**) 2 × 2 and 3 × 3, **B**) 2 × 1 and 3 × 1, and **C**) 1 × 2 and 1x3, **D**) PANI_ref.

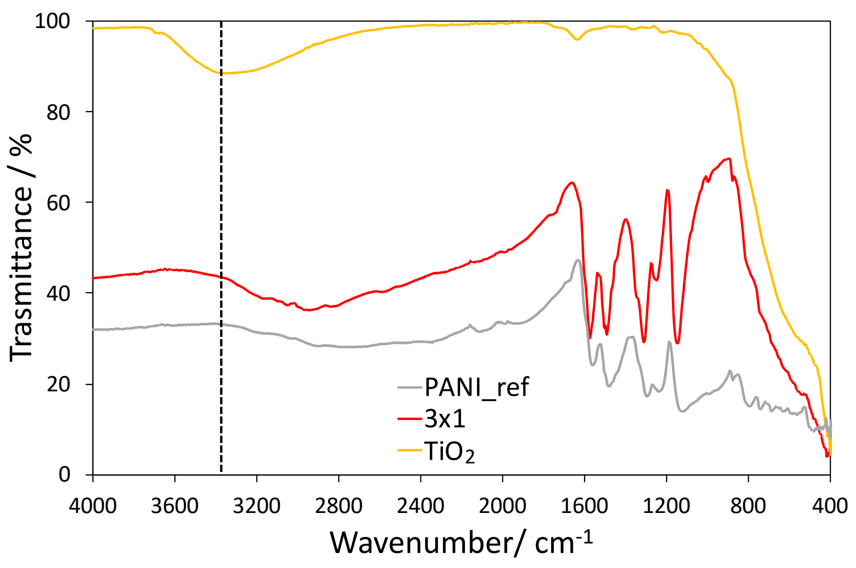

Figure S3. FTIR spectrum of 3 × 1 composite compared to those of PANI_ref and TiO_2: the vertical line is added only as a guide for the eyes.

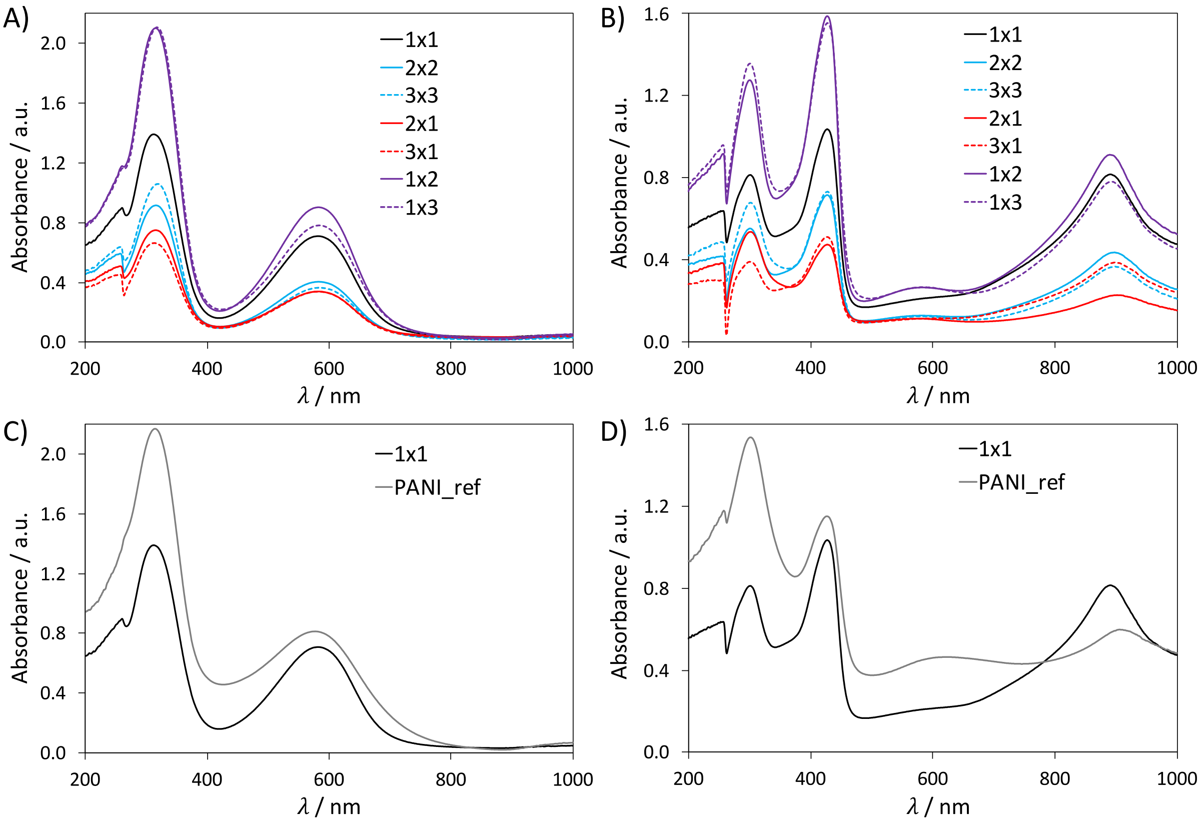

Figure S4. UV-vis spectra of all composites acquired **A**) in DMF solution and **B**) in DMF acidified with HCl; UV-vis spectra of PANI_ref compared to 1 × 1 sample **C**) in DMF and **D**) in DMF with HCl.

Table S2. Dependence of the H1/H2 ratio from the H_2O_2/aniline dimer molar ratio.

Sample	H_2O_2/Dimer	H1/H2
**1x1**	1	1.96
**2x1**	1	2.23
**3x1**	1	1.97
**2x2**	2	2.26
**1x2**	2	2.34
**3x3**	3	2.92
**1x3**	3	2.69

Table S3. Proposed structures attributed to main ESI-MS peaks.

	**m/z**
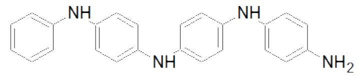	366
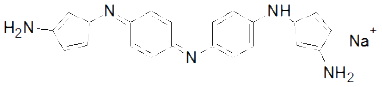	378
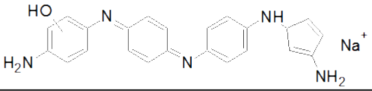	406
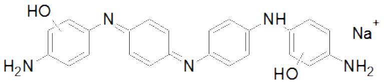	434
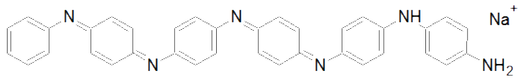	567
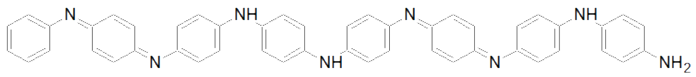	727
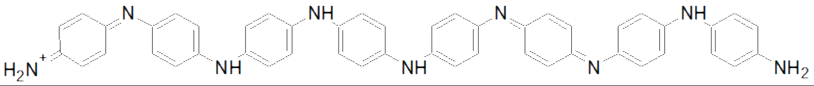	743
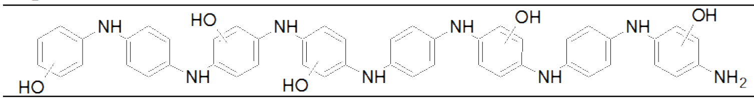	811
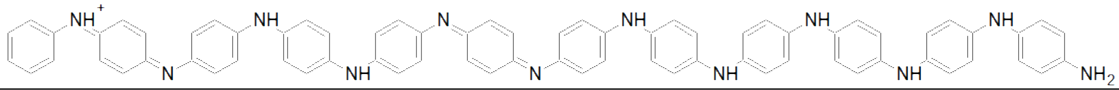	1092

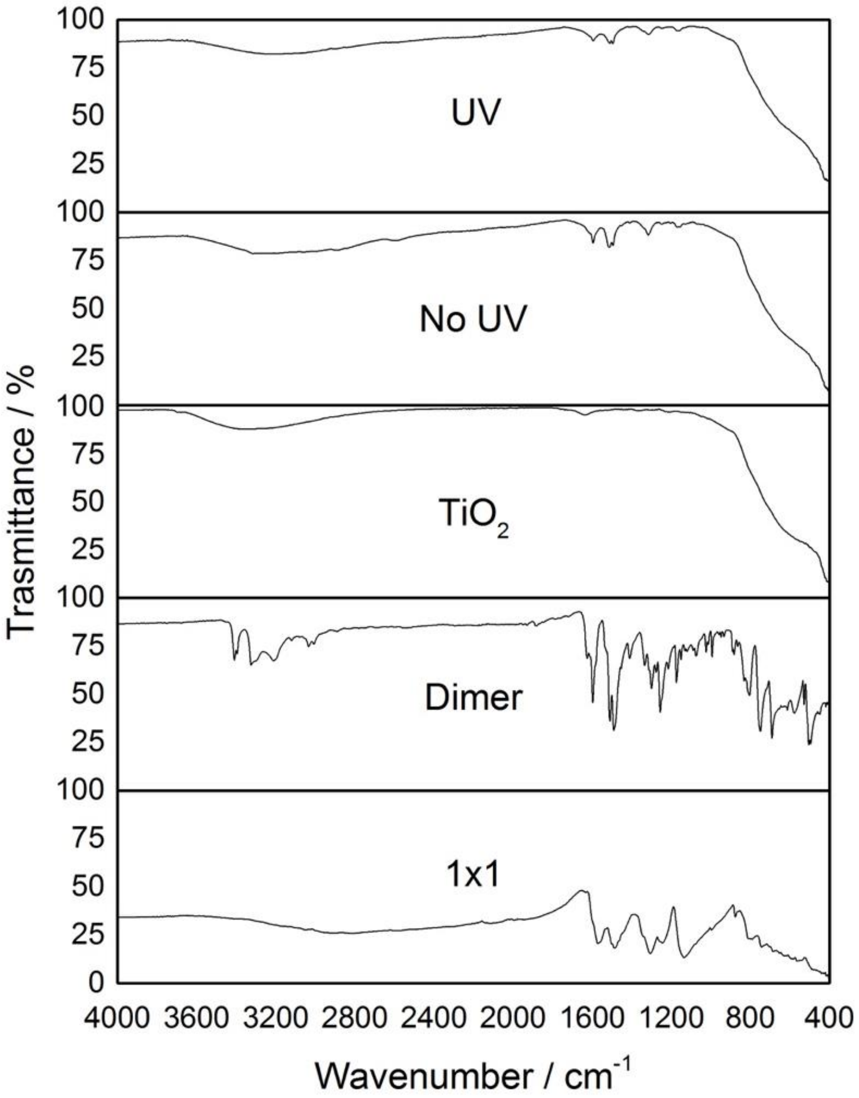

Figure S5. FT-IR spectra of the two tests studying the oligomerization step: tests under UV irradiation (UV) and in the dark (NoUV). The spectra of the pristineTiO_2, the aniline dimer and the 1 × 1 composite are reported for the sake of comparison.

Table S4. Surface elemental composition, determined from the XPS survey spectra, of NoUV, UV and pristine TiO_2 samples.

	**C/Ti**	**O/Ti**	**N/Ti**
**No UV**	2.6	2.6	0.19
**UV**	2.0	2.6	0.16
**TiO_2**	1.6	2.7	-

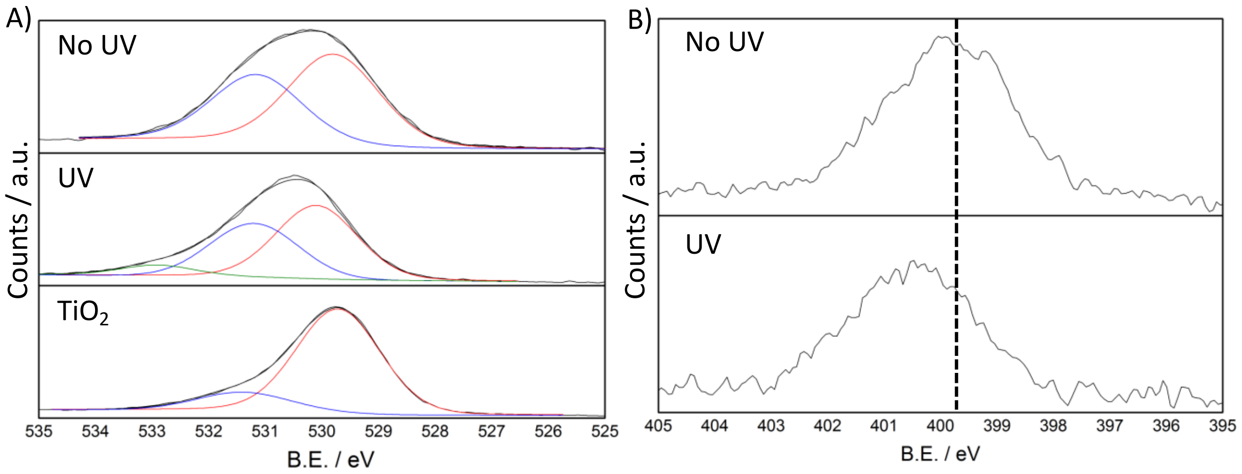

Figure S6. XPS spectra of the UV, NoUV and pristine TiO_2 samples in the **A**) O 1s and **B**) N 1s regions; the vertical line is added only as a guide for the eyes.

Table S5. Percentage of MO removed (± 2%) during sorption tests (50 mg composite, 20 mL of 50 ppm MO solution, 20 min).

**Sample**	**$MO_{removed}$ /%**
**1 × 1**	96
**2 × 2**	97
**3 × 3**	85
**2 × 1**	96
**3 × 1**	96
**1 × 2**	92
**1 × 3**	48

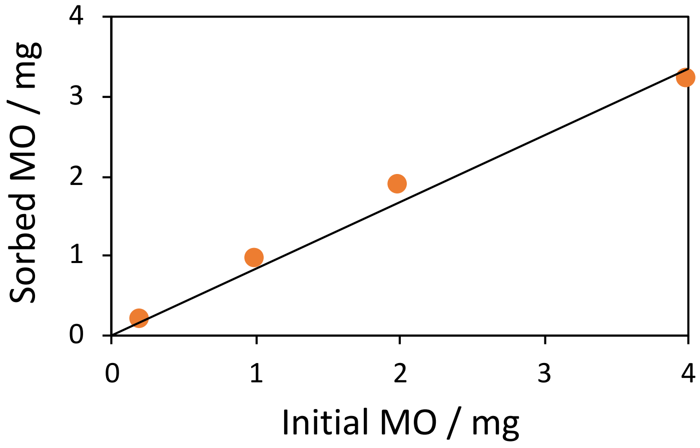

Figure S7. Sorbed MO amount as a function of the MO content of the initial solution for 1 × 1 sample.
